# Expression of Tight Junction Proteins and Cadherin 17 in the Small Intestine of Young Goats Offered a Reduced N and/or Ca Diet

**DOI:** 10.1371/journal.pone.0154311

**Published:** 2016-04-27

**Authors:** Kristin Elfers, Isabell Marr, Mirja R. Wilkens, Gerhard Breves, Marion Langeheine, Ralph Brehm, Alexandra S. Muscher-Banse

**Affiliations:** 1 Department of Physiology, University of Veterinary Medicine Hannover, Bischofsholer Damm 15/102, 30173, Hannover, Germany; 2 Institute of Anatomy, University of Veterinary Medicine Hannover, Bischofsholer Damm 15/122, 30173, Hannover, Germany; National Institute of Agronomic Research, FRANCE

## Abstract

Diets fed to ruminants should contain nitrogen (N) as low as possible to reduce feed costs and environmental pollution. Though possessing effective N-recycling mechanisms to maintain the N supply for rumen microbial protein synthesis and hence protein supply for the host, an N reduction caused substantial changes in calcium (Ca) and phosphate homeostasis in young goats including decreased intestinal transepithelial Ca absorption as reported for monogastric species. In contrast to the transcellular component of transepithelial Ca transport, the paracellular route has not been investigated in young goats. Therefore, the aim of the present study was to characterise the effects of dietary N and/or Ca reduction on paracellular transport mechanisms in young goats. Electrophysiological properties of intestinal epithelia were investigated by Ussing chamber experiments. The expression of tight junction (TJ) and adherens junction (AJ) proteins in intestinal epithelia were examined on mRNA level by *q*PCR and on protein level by western blot analysis. Dietary N reduction led to a segment specific increase in tissue conductances in the proximal jejunum which might be linked to concomitantly decreased expression of cadherin 17 mRNA. Expression of occludin (OCLN) and zonula occludens protein 1 was increased in mid jejunal epithelia of N reduced fed goats on mRNA and partly on protein level. Reduced dietary Ca supply resulted in a segment specific increase in claudin 2 and claudin 12 expression and decreased the expression of OCLN which might have been mediated at least in part by calcitriol. These data show that dietary N as well as Ca reduction affected expression of TJ and AJ proteins in a segment specific manner in young goats and may thus be involved in modulation of paracellular Ca permeability.

## Introduction

In ruminant feeding systems, low dietary nitrogen (N) contents are desirable for economic and environmental reasons to reduce feed costs and N output. Due to the rumino-hepatic cycle, ruminants like goats are able to recycle N effectively and to maintain N supply for microbial protein synthesis during low dietary N intake. For a sufficient synthesis of microbial protein by ruminal microbes, besides a certain amount of N sources, an adequate supply of dietary energy and macrominerals must be guaranteed. If these conditions are met, microbial protein accounts for the main protein supply of the host and hence, ruminants were thought to cope easily with a reduced dietary N content, in contrast to monogastric species which showed severe changes in mineral, especially calcium (Ca) homeostasis when consuming a protein reduced diet [1–4].

Own previous studies have shown that dietary N reduction affected overall Ca homeostasis in young goats leading to a decrease in plasma calcitriol concentrations and intestinal transcellular Ca absorption [5, 6]. Additionally, though a single dietary Ca reduction stimulated intestinal Ca absorption in young and adult goats [5, 7]; a concomitant reduction in N supply eliminated this stimulating effect in young goats [5, 8]. Diminished transcellular Ca absorption in goats fed an N reduction or an N and Ca reduction was associated with reduced plasma calcitriol concentrations as the main regulator of transcellular Ca transport in the small intestine [5].

Besides transcellular transport, intestinal Ca absorption can also be mediated by a paracellular pathway, whereby the latter one is mainly dependent on the electrochemical gradient [9]. On a molecular basis, paracellular transport processes are mediated through the junctional complex consisting of for example tight junctions (TJ), which encircle the apical and lateral membranes of enterocytes building charge and size selective pores [10]. The most important components of TJ proteins are claudins (CLDN) with a group of “tightening” and a group of permeability mediating members [11]. The latter group includes the CLDN2 and CLDN12, building cation-selective pores highly permeable for Ca [12–14]. Additionally, occludin (OCLN), which was the first identified TJ protein and zonula occludens protein 1 (ZO1) are members of the TJ. The OCLN mediates epithelial permeability for large molecules and its expression was shown to be coupled to the expression of Ca permeable CLDN2, therefore contributing to overall Ca permeability [15, 16]. The ZO1 is a stabilising protein providing the connection to the cytoskeleton of the cell and hence ensuring the correct assembly of TJ [17]. Furthermore, adherens junctions (AJ), mainly composed of cadherins (CDH) including CDH17 are localised at the junctional complex in the intestine and represent structures responsible for the mechanical strength due to their adhesive properties [18, 19].

From studies with monogastric species and cell cultures, evidence arose that expression of the above mentioned structures involved in paracellular Ca transport are regulated by calcitriol: In Caco-2 cells treatment with calcitriol led to a significant increase in mRNA and protein expression of CLDN2 and CLDN12 resulting in increased flux rates of radioactive labelled Ca [14]. Furthermore, a calcitriol-dependent up regulation of CLDN2 and CLDN12 in enterocytes was shown in vivo [20]. Decreased expression of CDH17 mRNA was measured in mice’s and rats’ intestinal tissues during low Ca intake and resulting high plasma calcitriol concentrations [21, 22] or after calcitriol application [23]. Additionally, studies in Caco-2 cells showed depressed CDH17 protein expression after treatment with calcitriol [22]. So far nothing is known about altered paracellular Ca transport based on changed TJ protein expression and the potential involvement of these changes affecting Ca homeostasis during dietary protein and N restriction in monogastric species and ruminants, respectively.

Based on the recorded changes in transcellular Ca absorption in young goats receiving an N and/or Ca reduced diet, the first hypothesis of the current study was that paracellular Ca transport is also affected by an N and/or Ca reduced feeding regime in young goats. Secondly, due to the important role calcitriol plays regarding modulation of TJ protein expression in monogastric species, it was hypothesised that potential changes in paracellular Ca transport in young goats on an N and/or Ca reduced diet, were based on altered calcitriol concentrations leading to changes in the expression of TJ proteins.

Hence, the aims of the present study were to characterise the electrophysiological properties of caprine intestinal epithelia and to determine the expression levels of CLDN2, CLDN12, OCLN, ZO1 and CDH17 in different sections of the small intestine of young goats fed an N and/or Ca reduced diet. With the present study, the expression of the mentioned structures as affected by the applied feeding strategy was successfully shown in the caprine intestine for the first time.

## Materials and Methods

The experimental conditions and the animal treatment were approved and the conduct thereof was supervised by the Animal Welfare Commissioner of the University of Veterinary Medicine Hannover according to the German Animal Welfare Law (permit number:33.12-42502-04-13/1045).

### Animals and feeding regimes

Animals, dietary ingredients and exact conditions of feeding regimes have been published recently by Elfers et al. [5]. Briefly, a total of 26 male coloured German goats aged eight weeks were randomly divided into four feeding groups with six to seven animals (n) housed together in each group. All groups received isoenergetic feeding. Depending on the feeding group the animals were fed three times a day and received either an adequate N and Ca supply (control group [N+/Ca+], 21% crude protein (CP), 1% Ca, n = 7), a reduced N supply ([N-/Ca+], 8% CP, 1% Ca, n = 6), a reduced Ca supply ([N+/Ca-], 22% CP, 0.4% Ca, n = 6) or a combined N and Ca reduction ([N-/Ca-], 8% CP, 0.4% Ca, n = 7) for 6–8 weeks. Chopped wheat straw was offered at 25% of the concentrate weight. Water was freely available. The animals were weighed weekly.

### Tissue sampling

At the end of the feeding period (after six to eight weeks) one animal per day was sacrificed by exsanguination after captive bolt stunning always at the same time in the morning and in a group-alternating manner.

Intestinal segments of the proximal jejunum, mid jejunum and ileum were taken from the intestinal tract within 5 min after exsanguination, opened along the mesenteric line and rinsed with ice-cold saline (0.9%, w/v) and were kept in a glucose-containing Krebs-Henseleit buffer solution that was aerated with carbogen (95% O_2_-5% CO_2_) before being mounted in Ussing chambers. For RNA isolation and preparation of crude membranes the mucosa of all three intestinal segments was stripped off, frozen in liquid N_2_ and stored at -80°C till further preparation.

For immunohistochemical examination, samples of mid jejunal epithelia of goats of the (N+/Ca+) group were fixed in 4% phosphate-buffered formaldehyde solution (Roti-Histofix, pH 7.0, Roth, Karlsruhe, Germany) or Bouin’s solution (10% formaldehyde, 4% picric acid, 5% acetic acid) for further preparation.

### Electrophysiological studies

After stripping the serosal and muscle layers from the mucosal layers, intestinal epithelia were mounted in Ussing chambers with an exposed serosal area of 1.13cm^2^. Tissues were incubated on both sides with a 38°C warm buffer solution containing (mM) 113.6 NaCl, 5.4 KCl, 1.2 MgCl_2_.6H_2_O, 21.0 NaHCO_3_, 1.2 CaCl.2H_2_O, 1.2 NaHPO_4_.2H_2_O, 1.2 mannitol and 0.01 indomethacin. Additionally, the serosal buffer contained 10.0 mM Glucose and 7.0 mM HEPES, whereas 20.0 mM HEPES was added to the mucosal buffer. The solutions were continuously aerated with carbogen; the pH was adjusted to 7.4. The potential differences, tissue conductances (G_t_) and short circuit currents (I_sc_) were continuously monitored by a computer-controlled voltage clamp device (Mussler Scientific Instruments, Aachen, Germany). Basal G_t_ and I_sc_ for proximal and mid jejunal epithelia were calculated from means over time in control chambers where no substances except for forskolin were added. In the case of ileal epithelia, basal G_t_ and I_sc_ were calculated from means over time starting after equilibration until adding blocker (see below). All experiments were carried out under short-circuited conditions. The 2,4,6 triaminopyrimidin (TAP) was used to characterise the paracellular route as it was done for blockage of gallbladder TJ channels [24]. The exact mode of action of TAP is currently not known, but it was hypothesised that it either induces conformational changes of TJ or alters TJ permeability by direct binding to certain binding sites within the TJ [24]. Approximately 1 h after starting the experiments, G_t_ and I_sc_ were determined before and after adding 20 mM TAP to the mucosal side of the chamber. The concentration of TAP was chosen according to data from literature [25, 26]. In addition to the continuous plotting of G_t_ and I_sc_ for monitoring tissue viability, the secretory response of intestinal epithelia to forskolin (50 μM, added to the serosal side) and glucose (10 mM, added to the mucosal side) were used to induce second messenger mediated stimulation of chloride secretion and hence changes in I_sc_ (forskolin) or carrier-mediated (glucose) changes in I_sc_, respectively, to confirm the viability of the epithelia at the end of the experiments.

### Total RNA isolation and reverse transcription

Total RNA was isolated using the RNeasy Plus Kit (Qiagen, Hilden, Germany) according to the manufacturer’s protocol. The concentration, quality and integrity of isolated RNA were determined by spectrophotometric measurements (BioPhotometer plus, Eppendorf AG, Hamburg, Germany) and by using an RNA 6000 nanoassay for an Agilent 2100 Bioanalyzer (Agilent Technologies, Böblingen, Germany). Integrity of the isolated RNA of all intestinal epithelia which were used for *q*PCR, expressed as mean RNA integrity number was between 8.9 (proximal jejunum), 8.7 (mid jejunum) and 8.8 (ileum), respectively (data not shown).

The reverse transcription of 200 ng RNA was done by using TaqMan Reverse Transcriptions Reagents Kit (Applied Biosystems, Roche Molecular System, Darmstadt, Germany) according to the manufacturer’s protocol.

### Intestinal expression of CLDN2, CLDN12, CDH17, OCLN and ZO1 mRNA

Caprine gene specific TaqMan primers and probes were synthesised by TIB MOLBIOL (Berlin, Germany; Table 1) to quantify the mRNA expression of GAPDH, CLDN2 and CLDN12. Each reaction mixture of 20 μl contained TaqMan Universal PCR Master Mix (Applied Biosystems), 16 ng cDNA, 300 nM specific primers and 100 nM of specific probe. The PCR product was amplified (50°C, 2 min; 95°C, 10 min; 40 cycles of 95°C, 15 s and 60°C, 1 min) and analysed on a real-time PCR cycler (CFX96TM, Bio-Rad, Munich, Germany). For quantifying CDH17, OCLN and ZO1 mRNA expression SYBR Green^®^ PCR assays were performed with specific primers from TIB MOLBIOL (Berlin, Germany; Table 2). Reaction mixtures (20 μl) contained KAPA SYBR FAST Universal Master Mix (PEQLAB Biotechnologie GmbH, Erlangen, Germany), 200 nM specific primers and 16 ng reverse transcribed cDNA. PCR products were amplified (95°C, 3 min; 40 cycles of 95°C, 10 s and 60°C, 30 s) and detected on a real-time PCR cycler (CFX96TM, Bio-Rad). The thermal profile for determining the melting curve began with an incubation of 10 min at 55°C with a gradual increase in temperature (0.5°C per 10 s). Absolute copy numbers were determined using calibration curves generated with cloned PCR fragment standards [27]. Specificity of the amplicons was verified by sequencing (GATC, Konstanz, Germany) and using NCBI Blast (http://blast.ncbi.nlm.nih.gov/Blast.cgi). Expression of genes of interest was normalised to GAPDH as a constant expressed housekeeping gene. Reactions were performed in duplicate and included a no-template water control.

**Table 1:** Primers and probes used for TaqMan assays.

Gene	Primers and probes (5′→3′)	Source	Efficiency	R^2^
GAPDH	sense: CAAGGTCATCCATGACCACTTT	[27]	99.5%	0.98
	antisense: CGGAAGGGCCATCCACA			
	FAM-CTGTCCACGCCATCACTGCCACCC-TMR			
CLDN2	sense: CCAAAGACAGAGTGGCGGT	this study	106.9%	0.99
	antisense: TCAAATTTCATGCTGTCAGGCAC			
	FAM-TCCTGGGCTTCATCCCYGTTGC-BBQ			
CLDN12	sense: GCTGCTCTGCCTCATCGG	this study	105.2%	0.99
	antisense: GCAGCCYGCACTATTGACCA			
	FAM-TGTGTAACACGGCCTTCAGGTCCTC-BBQ			

**Table 2:** Primers used for SYBR assays.

Gene	Primers and probes (5′→3′)	Source	Efficiency	R^2^
CDH17	sense: CACCCTTTTGGTCATCGGTAT	this study	95.1%	0.97
	antisense: CATCAGTTTCTCAGAGGCTTGACT			
OCLN	sense: CTCGTCTGGATAAAGAACTGGATGA	this study	100.7%	0.99
	antisense: CTCGTCTGGATAAAGAACTGGATGA			
ZO1	sense: CTCAGTACAGCCAGGGTGCT	this study	108.1%	0.99
	antisense: TCCGGTTTGGACACTAATGAGTT			

### Intestinal expression of CLDN2, CLDN12, OCLN and ZO1 protein

In order to prepare crude membrane fractions of the proximal jejunum, mid jejunum and the ileum the protocol from Wilkens et al. [28] was used. Protein concentrations of all preparations were determined by the Bradford method (Bio-Rad).

For separation by SDS-PAGE (12% SDS gel for CLDN2 and CLDN12; 8.7% SDS gel for OCLN) 15μg of crude membrane fractions were incubated in a loading dye containing 5% dithiothreitol (DTT) and were transferred to nitrocellulose membranes (GE Healthcare, Fribourg, Germany) using a tank blotting system (Bio-Rad). In the case of ZO1, 100 μg of crude membrane fractions were incubated for 20 min at 70°C followed by SDS-PAGE (6% SDS gel) and transferred onto nitrocellulose membranes using a blotting buffer containing 0.05% SDS which allows large proteins to dissolve out of the gel. Membranes were blocked for 1 h (OCLN) or 2 h (CLDN2, CLDN12, ZO1), respectively in PBS containing 0.1% Tween 20 (PBST) and 5% fat-free milk powder. Primary antibodies were diluted in PBST containing 3% bovine serum albumin (BSA) in the case of CLDN2 and CLDN12 (mouse monoclonal anti-claudin 2, catalogue number 32–5600, dilution 1:1000; rabbit polyclonal anti-claudin 12, catalogue number 38–8200, dilution 1:1000, both from Invitrogen; Life Technologies GmbH, Darmstadt, Germany) or in PBST in the case of OCLN and ZO1 (rabbit polyclonal anti-occludin, catalogue number ABT146, dilution 1:2000; rat monoclonal anti-ZO1, catalogue number MABT11, dilution 1:100; both from Merck Millipore, Darmstadt, Germany) and incubated at 4°C overnight. Specificity of the OCLN antibody in goats was validated by cross reactivity with bovine tissue reported by the manufacturer.

Immunodetection of electrotransferred proteins was performed according to standard procedures. After washing with PBST and incubating with the corresponding secondary antibody, bound antibody was visualised by using enhanced chemiluminescence (SuperSignal, Thermo Fisher Scientific) according to the manufacturer’s protocol and ChemiDoc system (Bio-Rad).

Quantification of protein amounts was carried out by using the Quantity One software 4.4 and Image Lab 5.2.1 software (Bio-Rad). Values of investigated proteins were normalised to the amount of ß-actin (anti-ß-actin, AC-15, Sigma Aldrich) or villin (anti-villin, CTS192 NatuTec, Frankfurt, Germany) as internal standards with a constant level of expression.

### Immunohistochemistry of CLDN2 in mid jejunal epithelia

For exact localisation of CLDN2 protein in caprine intestinal epithelia exemplarily immunohistochemical staining of mid jejunal epithelia of goats of the (N+/Ca+) group was performed. Therefore, from paraffin-embedded segments 4 μm-thick sections were cut. During deparaffinisation and rehydration, H_2_O_2_ was used for blocking endogenous peroxidases. Sections were pretreated with EDTA buffer and subsequently blocked with BSA for 20 min. Incubation with the primary antibody (anti CLDN2, 1:2000; Invitrogen) was carried out over night at 4°C. Afterwards, sections were incubated with the corresponding secondary antibody (HRP anti-mouse, ready-to-use; EnVision, Dako, Hamburg, Germany) for 30 min. Immunoreactivity was visualised by diaminobenzidine (DAB; EnVision, Dako). Finally, sections were counterstained with hematoxylin. A negative control was performed by using the same protocol without incubation with the primary antibody.

A Zeiss Axioskop microscope (Carl Zeiss, Oberkochen, Germany, Zeiss Axioskop) was used to view the sections and images were made with a DP70 digital camera (Olympus, Hamburg, Germany, DP70).

### Statistical analysis

All values were given as means ± SEM. All data were tested for normal distribution by Kolmogorov-Smirnov test and analysed by ordinary two-way ANOVA with two fixed factors (N and Ca) followed by Tukey’s multiple comparisons test. A paired t-test was performed to determine differences between the values of I_sc_ or G_t_ before and after adding TAP in the electrophysiological studies. The *P* < 0.05 was considered as statistically significant, whereas *P* < 0.10 was assumed to be a trend. All statistical analyses were performed using GraphPad Prism 6.05 (GraphPad Software, San Diego, CA, USA, www.graphpad.com).

## Results

### Intake, body weight and daily weight gain

Daily dry matter (DM), concentrate, N, Ca and phosphorus (P) intake on a per-animal basis were calculated from group mean values. Mean daily DM, concentrate, N, Ca and P intake as well as feed efficiency are presented as supporting information in (S1 Table). Initial and final body weight as well as daily weight gain is shown in Table 3. These data are shown in order to characterise the experimental model and have recently been published by Elfers et al. [5].

**Table 3:** Initial and final body weight as well as daily weight gain of growing goats receiving an N and/or Ca reduced diet.

Item	N+/Ca+	N-/Ca+	N+/Ca-	N-/Ca-	*P* values of two-way ANOVA		
					N reduction	Ca reduction	N x Ca interaction
n	7	6	6	7			
Initial body weight (kg)	15.6 ± 0.68	15.5 ± 1.07	16.6 ± 1.00	16.7 ± 0.78	0.97	0.22	0.91
Final body weight (kg)	23.93 ± 1.78	21.17 ± 0.66	23.58 ± 0.92	21.07 ± 1.05	0.04	0.86	0.92
Body weight gain (kg/d)	0.18 ± 0.02	0.15 ± 0.01	0.16 ± 0.01	0.15 ± 0.02	0.24	0.55	0.62

Mean values ± SEM; n = number of animals; data have already been published by Elfers et al. [5]

### Influence of a reduced N and/or Ca diet on electrophysiological parameters of intestinal epithelia in young goats

In the following, results are only described if differences between the feeding groups were determined. All data are summarised in Table 4.

**Table 4:** Electrophysiological properties of proximal, mid jejunal and ileal epithelia of young goats during dietary N and/or Ca reduction.

Item	N+/Ca+	N-/Ca+	N+/Ca-	N-/Ca-	*P* values of two-way ANOVA		
					N reduction	Ca reduction	N x Ca interaction
n	7	6	6	7			
prox. jej.							
G_t_	20.31 ± 2.12	23.84 ± 1.44	19.05 ± 1.33	22.48 ± 1.15	0.04	0.42	0.98
I_sc_	0.59 ± 0.08	0.83 ± 0.05	0.52 ± 0.04	0.74 ± 0.13	0.01	0.36	0.89
mid jej.							
G_t_	22.13 ± 1.57^a^^,^^b^	25.87 ± 0.70^a^	22.36 ± 0.91^a^^,^^b^	20.29 ± 1.24^b^	0.49	0.04	0.03
I_sc_	-0.71 ± 0.38	-0.24 ± 0.32	-0.27 ± 0.27	-0.54 ± 0.29	0.77	0.83	0.26
ileum							
G_t_	16.13 ± 0.85	16.67 ± 0.83	16.45 ± 1.31	14.74 ± 0.79	0.55	0.41	0.25
I_sc_	1.68 ± 0.19	1.72 ± 0.18	2.10 ± 0.27	2.28 ± 0.45	0.74	0.12	0.83

I_sc_, short-circuit current in μeq/(cm^2^xh); jej., jejunum; G_t_, tissue conductance in mS/cm^2^; prox., proximal. Means ± SEM; n = number of animals.

^a,b^ Mean values within a row with dissimilar superscript letters were significantly different; Tukey’s multiple comparisons test (*P* < 0.05).

In the proximal jejunum both, basal I_sc_ and G_t_ were significantly increased due to N restriction (*P* = 0.01 and *P* = 0.04). In the mid jejunum, a significant interaction between N and Ca reduced feeding was detected for basal G_t_ (*P* = 0.03), leading to significantly reduced G_t_ in the (N-/Ca-)-group compared to (N-/Ca+)-group. In the ileum, basal I_sc_ was increased by trend in the Ca restricted groups (*P* = 0.12).

**Fig 1:**
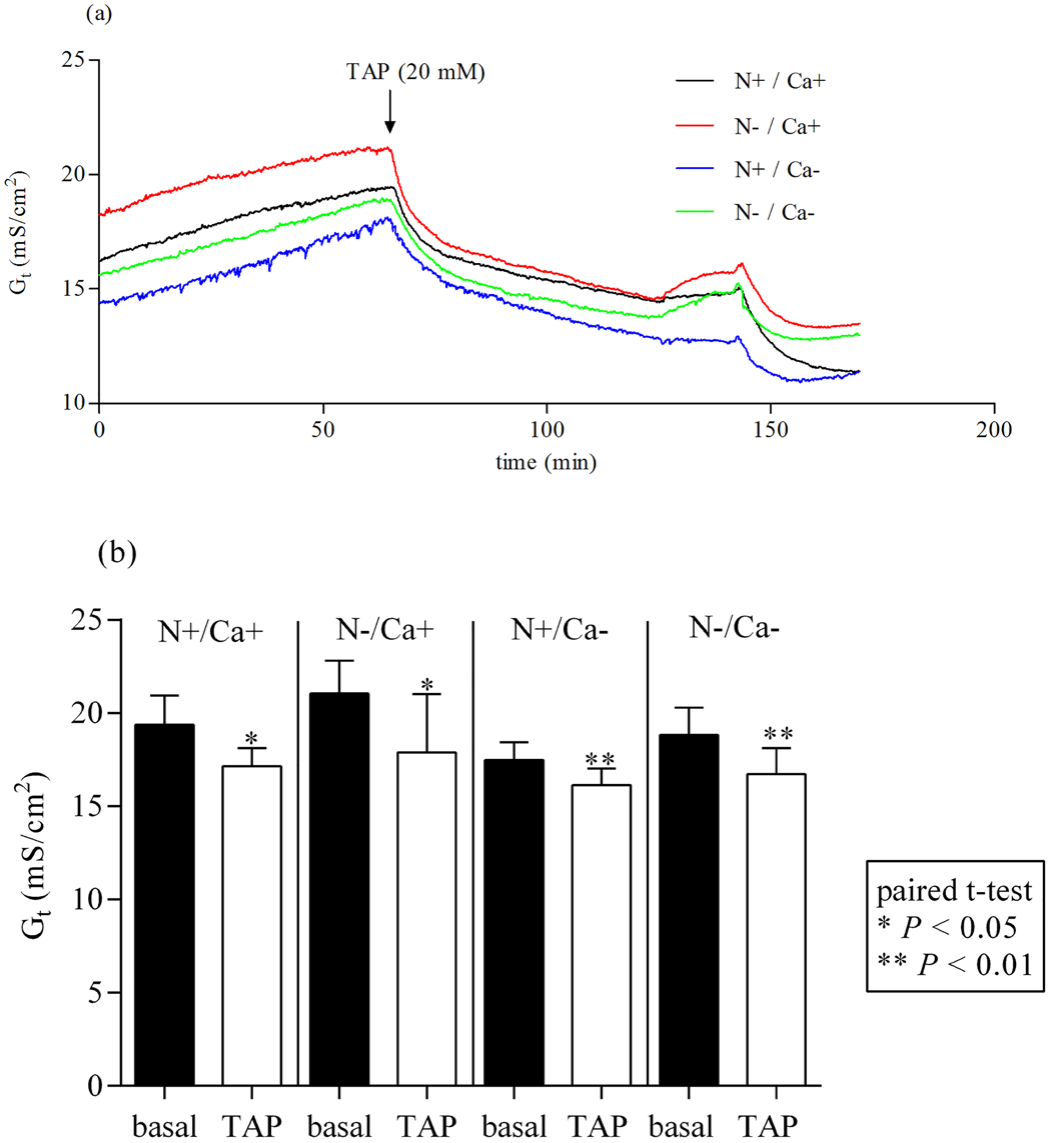
Effect of mucosal addition of 20 mM TAP in the proximal jejunum. TAP, 2,4,6 triaminopyrimidin. Effect on tissue conductance (G_t_) (a). Inhibition of G_t_ statistically quantified by paired t-test (b).

**Fig 2:**
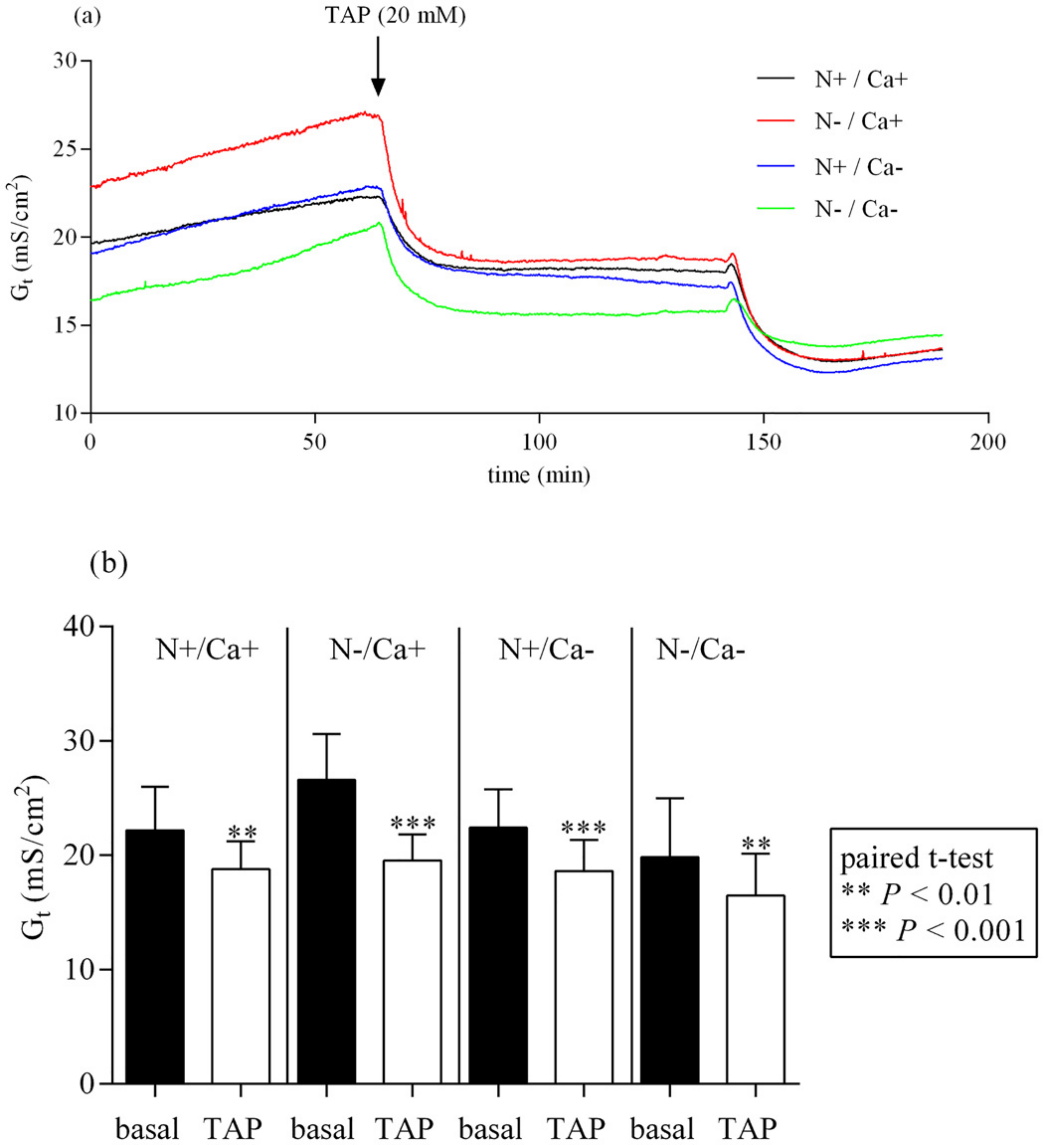
Effect of mucosal addition of 20 mM TAP in the mid jejunum. TAP, 2,4,6 triaminopyrimidin. Effect on tissue conductance (G_t_) (a). Inhibition of G_t_ statistically quantified by paired t-test (b).

After adding 20 mM TAP to the mucosal side of epithelia of the proximal jejunum, G_t_ decreased significantly in all feeding groups (Fig 1a and 1b) (*P* < 0.05 for [N+/Ca+] and [N-/Ca+] and *P* < 0.01 for [N+/Ca-] and [N-/Ca-]). The same effect could be detected in the mid jejunum irrespective of the feeding regime (Fig 2a and 2b) (*P* < 0.01 for [N+/Ca+] and [N-/Ca-] and *P* < 0.001 for [N-/Ca+] and [N+/Ca-]). The G_t_ in ileal epithelia after adding TAP and G_t_ in parallel measured control tissues without TAP did not change (data not shown).

### Influence of a reduced N and/or Ca diet on intestinal expression of CLDN2, CLDN12, CDH17, OCLN and ZO1 mRNA

All mRNA expression data are summarised in Table 5.

**Table 5:** RNA expression of tight junction proteins normalised to GAPDH in the proximal and mid jejunum and ileum of goats fed an N and/or Ca reduced diet.

Item	N+/Ca+	N-/Ca+	N+/Ca-	N-/Ca-	*P* values of two-way ANOVA		
					N reduction	Ca reduction	N x Ca interaction
n	7	6	6	7			
CLDN2							
prox. jej.	0.14 ± 0.012	0.09 ± 0.015	0.13 ± 0.018	0.13 ± 0.018	0.16	0.39	0.27
mid jej.	0.044 ± 0.007	0.043 ± 0.003	0.062 ± 0.009	0.052 ± 0.007	0.47	0.06	0.53
ileum	0.22 ± 0.05	0.20 ± 0.05	0.39 ± 0.04	0.28 ± 0.08	0.27	0.05	0.46
CLDN12							
prox. jej.	0.00084 ± 0.69e-04^a^	0.001 ± 0.42e-04^a^^,^^b^	0.0011 ± 0.85e-04^a^^,^^b^	0.0011 ± 0.47e-04^b^	0.08	0.04	0.21
mid jej.	0.0013 ± 0.0001	0.0016 ± 0.0002	0.0017 ± 0.0001	0.0015 ± 0.0001	0.58	0.25	0.08
ileum	0.0051 ± 0.0008	0.0046 ± 0.0008	0.0056 ± 0.0004	0.0058 ± 0.001	0.87	0.33	0.73
CDH17							
prox. jej.	0.067 ± 0.005 ^a^^,^^b^	0.047 ± 0.005^a^†	0.073 ± 0.007 ^b^	0.065 ± 0.004 ^a^^,^^b^	0.02	0.04	0.30
mid jej.	0.058 ± 0.004	0.058 ± 0.004	0.063 ± 0.006	0.068 ± 0.007	0.64	0.22	0.63
ileum	0.058 ± 0.009	0.046 ± 0.007	0.071 ± 0.008	0.051 ± 0.007	0.05	0.26	0.60
OCLN							
prox. jej.	0.0045 ± 0.0003	0.0046 ± 0.0005	0.0043 ± 0.0005	0.0047 ± 0.0004	0.46	0.94	0.73
mid jej.	0.0057 ± 0.0008^a^	0.0082 ± 0.0002^b^	0.0054 ± 0.0004^a^	0.0057 ± 0.0004^a^	0.01	0.01	0.05
ileum	0.003 ± 0.0005	0.003 ± 0.0006	0.003 ± 0.0005	0.003 ± 0.0004	0.66	0.59	0.64
ZO1							
prox. jej.	0.064 ± 0.0003	0.0053 ± 0.0005	0.0056 ± 0.0007	0.0059 ± 0.0005	0.42	0.85	0.20
mid jej.	0.005 ± 0.0003	0.0054 ± 0.0004	0.048 ± 0.0003	0.006 ± 0.0006	0.08	0.62	0.36
ileum	0.0061 ± 0.0007	0.0062 ± 0.001	0.007 ± 0.0007	0.0059 ± 0.0004	0.53	0.72	0.39

CDH17, cadherin 17; CLDN2, claudin 2; CLDN12, claudin 12; jej., jejunum; OCLN, occludin; prox., proximal; ZO1, zonula occludens protein 1. Means ± SEM; n = number of animals;

^†^ One animal was excluded from analysis to obtain Gaussian distribution.

^a,b^ Mean values within a row with dissimilar superscript letters were significantly different; Tukey’s multiple comparisons test (*P* < 0.05).

In the proximal jejunum there were no significant differences between the feeding groups regarding the CLDN2 mRNA expression. The Ca reduced fed goats tended to result in increased CLDN2 mRNA expression in the mid jejunum (*P* = 0.06) and significantly elevated CLDN2 mRNA expression in the ileum (*P* = 0.05).

The mRNA expression of CLDN12 was exclusively altered in the proximal jejunum with a trend towards increased expression due to dietary N reduction (*P* = 0.08) and a significantly increased expression due to dietary Ca reduction (*P* = 0.04). No changes in CLDN12 mRNA expression were detected in the mid jejunum and ileum.

Expression of CDH17 mRNA was significantly decreased in the proximal jejunum and ileum in N reduced fed goats (*P* = 0.02 and *P* = 0.05), while dietary Ca reduction led to increased expression levels in the proximal jejunum (*P* = 0.05). No differences of CDH17 expression were shown in the mid jejunum.

Regarding OCLN mRNA expression there was a significant interaction of dietary N and Ca reduction detectable in the mid jejunum (*P* = 0.05), leading to increased expression in the (N-/Ca+) group, while the (N-/Ca-) group showed numerically unaltered expression compared to (N+/Ca+). In the proximal jejunum and ileum, expression of OCLN remained unaffected.

While ZO1 mRNA expression was not modulated by any dietary intervention in the proximal jejunum and ileum, goats receiving N reduced feed showed a trend towards increased ZO1 mRNA expression in the mid jejunum (*P* = 0.08).

### Intestinal expression of CLDN2, CLDN12, OCLN and ZO1 protein

**Fig 3:**
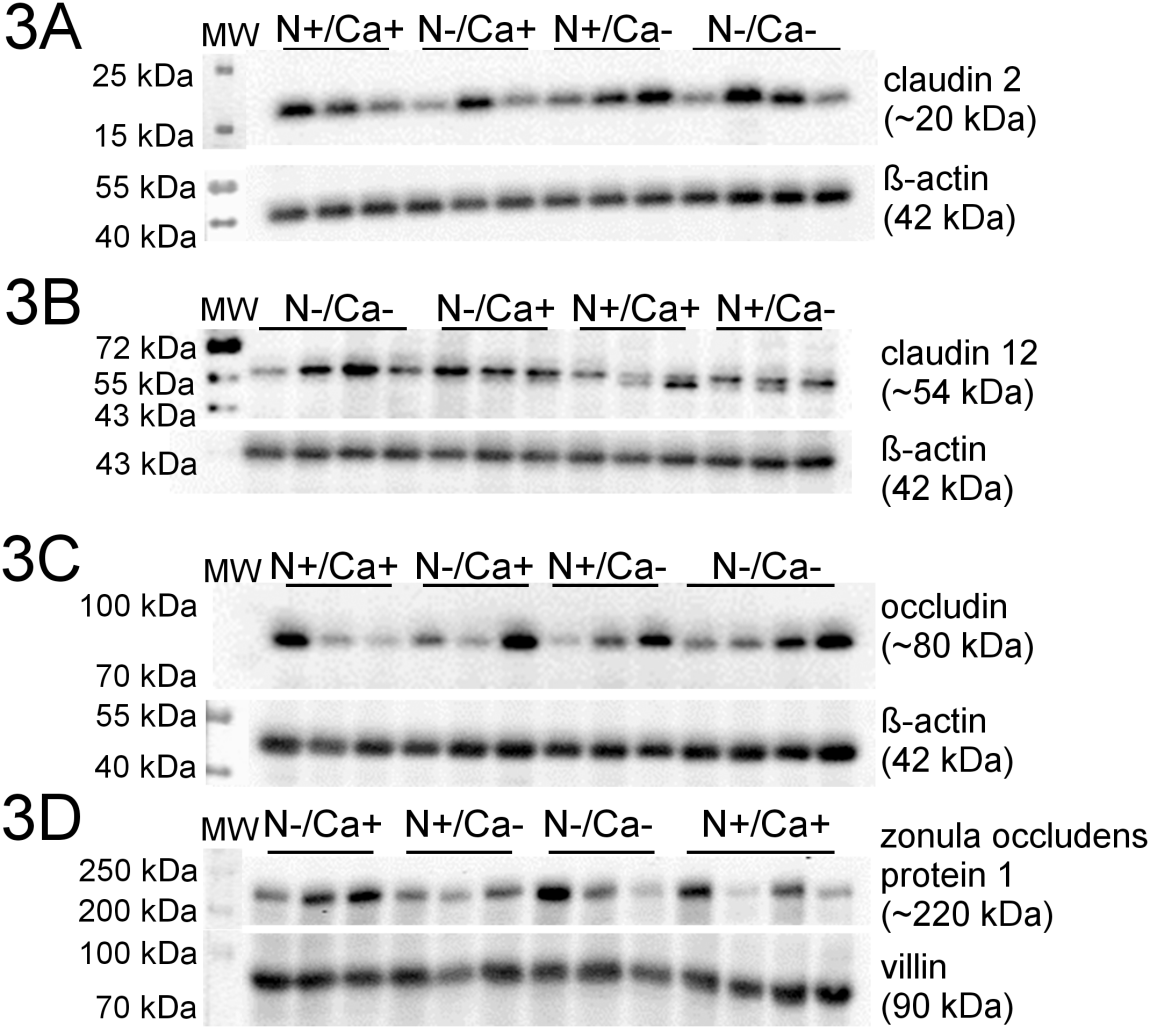
Representative signals of investigated proteins in the proximal jejunum of young goats receiving an N and/or Ca reduced diet. A: CLDN2; B: CLDN12; C: OCLN; D: ZO1.

**Fig 4:**
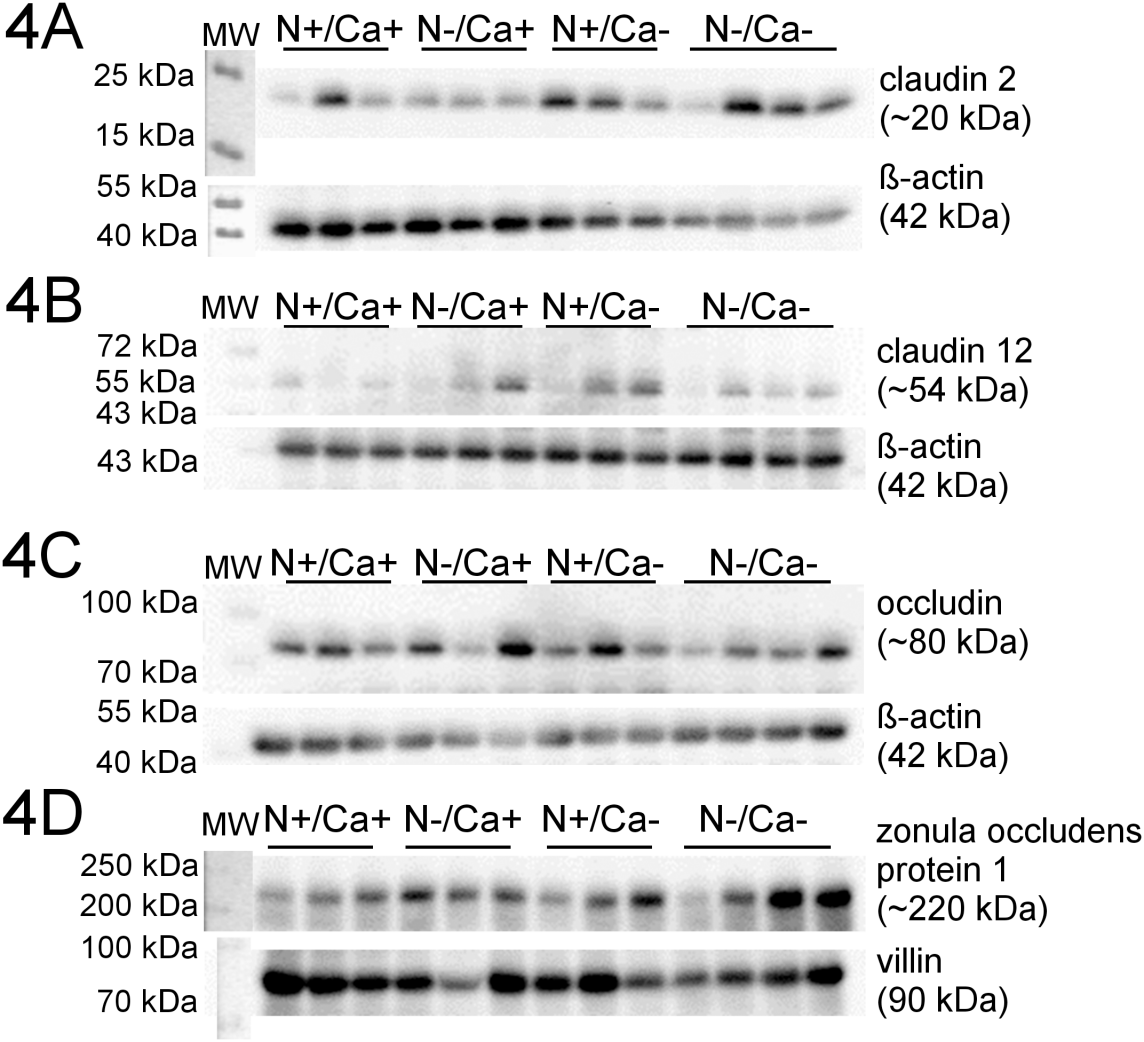
Representative signals of investigated proteins in the mid jejunum of young goats receiving an N and/or Ca reduced diet. A: CLDN2; B: CLDN12; C: OCLN; D: ZO1.

**Fig 5:**
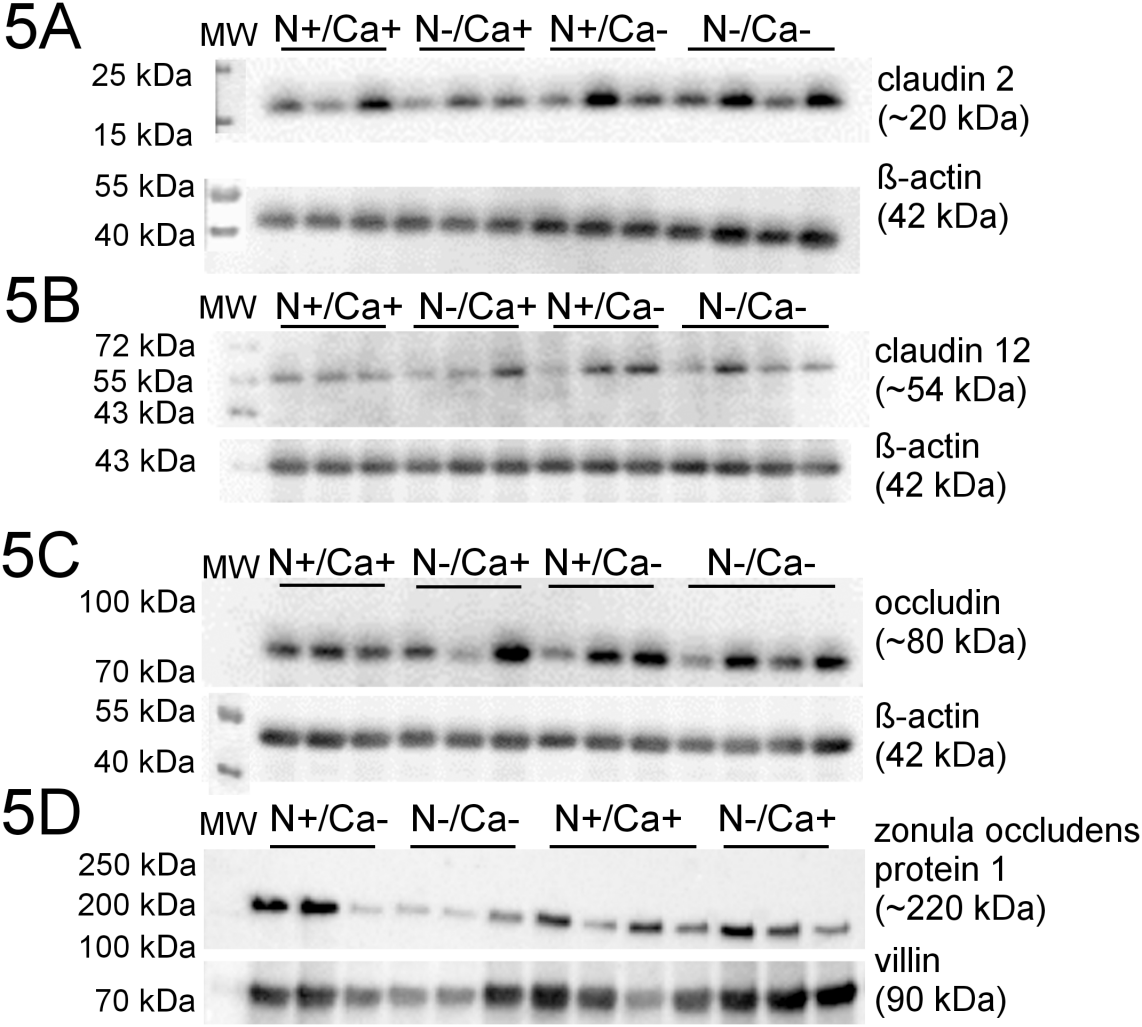
Representative signals of investigated proteins in the ileum of young goats receiving an N and/or Ca reduced diet. A: CLDN2; B: CLDN12; C: OCLN; D: ZO1.

Representative Western Blots of all examined proteins are shown in Figs 3, 4 and 5. Signals for proteins of interest were detected at expected molecular weight, ~20 kDa for CLDN2, ~80 kDa for phosphorylated OCLN and ~220 kDa for ZO1. For CLDN12 the homodimeric form (~54 kDa) was detected.

Results of protein expression are summarised in Table 6.

**Table 6:** Relative amounts of tight junction protein expression normalised to ß-actin or villin in the proximal and mid jejunum and ileum of goats fed an N and/or Ca reduced diet.

Item	N+/Ca+	N-/Ca+	N+/Ca-	N-/Ca-	*P* values of two-way ANOVA		
					N reduction	Ca reduction	N x Ca interaction
n	7	6	6	7			
CLDN2							
prox. jej.	0.66 ± 0.05	0.60 ± 0.09	0.68 ± 0.06	0.70 ± 0.10	0.75	0.44	0.57
mid jej.	0.72 ± 0.20	0.50 ± 0.09	0.71 ± 0.11	0.74 ± 0.11	0.50	0.41	0.37
ileum	0.67 ± 0.06	0.61 ± 0.08	0.68 ± 0.09	0.67 ± 0.03	0.54	0.64	0.74
CLDN12							
prox. jej.	0.51 ± 0.05	0.51 ± 0.04	0.45 ± 0.06	0.49 ± 0.04	0.66	0.37	0.61
mid jej.	0.39 ± 0.05	0.45 ± 0.09	0.42 ± 0.14	0.38 ± 0.07	0.85	0.85	0.59
ileum	0.43 ± 0.03	0.47 ± 0.05	0.44 ± 0.05	0.44 ± 0.05	0.65	0.84	0.65
OCLN							
prox. jej.	0.82 ± 0.14	1.34 ± 0.21	1.10 ± 0.16	1.10 ± 0.12	0.12	0.90	0.12
mid jej.	0.80 ± 0.10	0.78 ± 0.16	0.59 ± 0.08	0.45 ± 0.07	0.45	0.02	0.58
ileum	0.57 ± 0.03	0.59 ± 0.11	0.57 ± 0.06	0.50 ± 0.08	0.77	0.50	0.54
ZO1							
prox. jej.	0.43 ± 0.09	0.66 ± 0.06	0.49 ± 0.06	0.56 ± 0.12	0.11	0.85	0.40
mid jej.	0.49 ± 0.08	0.61 ± 0.15	0.44 ± 0.06†	0.90 ± 0.17	0.04	0.37	0.22
ileum	0.62 ± 0.12	0.55 ± 0.09	0.65 ± 0.13	0.57 ± 0.11	0.53	0.81	0.97

CLDN2, claudin 2; CLDN12, claudin 12; jej., jejunum; OCLN, occludin; prox., proximal; ZO1, zonula occludens protein 1. Means ± SEM; n = number of animals;

^**†**^ One animal excluded from analysis to obtain Gaussian distribution.

No alterations of protein expression of CLDN2 and CLDN12 were measured in the three intestinal segments investigated. In the mid jejunum, a significantly decreased protein expression of OLCN in the Ca reduced fed goats was detected (*P* = 0.02), while neither in the proximal nor in the ileum were changes seen in OCLN protein expression. A likewise increased protein expression confined exclusively to the mid jejunum of N reduced fed goats was detected in the case of ZO1 (*P* = 0.04).

### CLDN2 staining in mid jejunal epithelia

**Fig 6:**
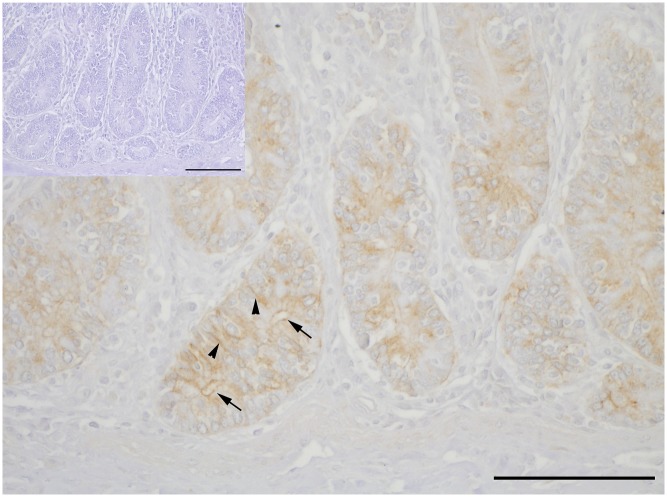
Representative single immunohistochemistry for CLDN2 in mid jejunal epithelial paraffin sections of (N+/Ca+) goats. CLDN2 is detectable apically (arrows) and in lateral membranes of enterocytes (arrowheads) in crypts. No unspecific staining was detected in the negative control (insert). Scale bars: 100 μm.

Clear immunopositive staining for CLDN2 was found in the apical and lateral membranes of epithelial cells in crypts of the mid jejunum of goats in the (N+/Ca+) group (Fig 6). Negative controls, without primary antibody incubation, were immunonegative (insert in Fig 6).

## Discussion

The aim of the present study was to evaluate effects of a dietary N or Ca reduction alone or in combination on electrophysiological properties of caprine intestinal epithelia and expression patterns of TJ and AJ proteins in the small intestine of young goats and the potential impact on paracellular Ca transport. It could be shown for the first time that dietary N reduction modulated the expression patterns of CDH17 and ZO1 expression on mRNA and partly on protein level in the upper part of small intestines in young goats. This might have induced changes in intestinal epithelial permeability, which was reflected by increased G_t_ at least in the proximal jejunum, and might be one reason for imbalances of Ca homeostasis seen in these animals. In contrast, dietary Ca reduction led to increased expression of Ca-permeable CLDN 2 and CLDN12 in the proximal and mid jejunum and ileum, respectively, probably contributing to an overall enhanced intestinal Ca absorption. Concomitantly decreased OCLN expression in the mid jejunum during dietary Ca reduction might have been coupled with the increased CLDN expression in a regulatory manner.

Limitations of the present study were the group feeding of the animals and a reduction in final body weights of the N reduced fed goats. Nevertheless, energy supply for all animals independent off feeding group met the recommendations of the GfE and was hence sufficient. Furthermore, continuous weight gain and unaffected plasma triiodothyronine (T3) concentrations [5] indicated sufficient energy intake of all goats, with T3 representing an energy-dependent hormone which dropped due to energy deprivation in adult sheep [29].

In the current study with the inhibiting effect of the TJ-blocker TAP on G_t_ in the proximal and mid jejunum in all feeding groups (Figs 1 and 2) the importance of paracellular transport processes in the caprine intestine was emphasised. In the mid jejunum, the inhibition of G_t_ was significantly greater in the N reduced fed goats (data not shown), probably indicating pronounced paracellular transport processes during this feeding regime. This was also supported by numerically highest serosal to mucosal flux rates of Ca in the N reduced fed goats which were mediated by the paracellular route revealed by correlation with the mannitol flux rates [5] as a functional indicator for paracellular permeability [30].

The inhibiting effect of TAP on total transepithelial conductance has already been shown in the toad gallbladder, this being due to a decreased permeability for Na^+^ and other cations like lithium and caesium [24]. In the current study, a feeding-independent, numerically (not statistically significant) inhibiting effect of mucosal added TAP on mucosal to serosal Ca flux rates (J_ms_Ca) could be detected in the mid jejunum (data not shown), indicating that TAP is a potent inhibitor for paracellular cation transport including Ca permeability in caprine intestinal epithelia as described by Moreno and coworkers for the toad gallbladder [24].

On a molecular basis paracellular transport is mediated by TJ proteins, including CLDN, OCLN and ZO proteins, whereby CLDN2 and CLDN12 built Ca specific permeable channels [12, 14, 20]. Increased mRNA expression of CLDN2 in the mid jejunum and ileum and increased mRNA expression of CLDN12 in the proximal jejunum of Ca reduced fed goats shown in the current study, might have enhanced paracellular Ca absorption and might therefore contribute to the overall increased Ca absorption measured in these goats [5]. Both, CLDN2 and CLDN12 mRNA as well as protein expression were shown to be increased by calcitriol in cell culture [14]. Hence, increased mRNA expression in goats on the Ca reduced diet was most likely mediated by increased plasma calcitriol concentrations measured in these goats [5]. Interestingly, mice with a knock-down of the Ca binding protein D_9K_ (CaBP_D9K_) necessary for transcellular intestinal Ca transport compensated for the severely impaired transcellular Ca absorption with increased expression of Ca permeable CLDNs, including CLDN2, underlying the tight connection between trans- and paracellular Ca transport in monogastric species [31].

In contrast, N reduced fed goats in the present study, which also showed decreased CaBP_D9K_ expression and therefore low transcellular Ca absorption [5], were obviously not able to compensate for the insufficient transcellular Ca absorption by increased expression of CLDN2 or CLDN12. Underlying mechanisms how low dietary N might prevent this compensation seen in monogastric species are unknown at present.

The non-classical cadherin CDH17, also known as liver-intestine cadherin due to the first finding of expression in these organs, mediates cell-cell adhesion in a Ca-dependent way [32], but in contrast to classical cadherins independently from the cytoskeleton [33]. In N reduced fed goats in the present study CDH17 mRNA expression was significantly reduced in the proximal jejunum which potentially led to impaired cell-cell adhesion. This might have mediated the significant increase in G_t_ in the proximal jejunum of N reduced fed goats. In addition, increased unidirectional serosal to mucosal Ca flux rates in the proximal jejunum of N reduced fed goats [5] were probably based on reduced cell adhesion and therefore enhanced paracellular flux of Ca into the intestinal lumen. This pronounced loss of Ca into the intestine in combination with reduced transcellular Ca absorption probably led to the significantly decreased plasma Ca concentrations measured in the N reduced fed goats [5]. In contrast, dietary Ca reduction led to stimulated CDH17 mRNA expression in the proximal jejunum, possibly marking a compensation of decreased expression of other (classical) cadherins not investigated in the current study, to ensure sufficient cell-cell adhesion [33] and therefore to avoid loss of solutes, especially of Ca into the lumen. In rat and mouse intestine, calcitriol led to a decrease in CDH17 mRNA expression [22, 23]. Against the background of decreased calcitriol concentrations in N reduced fed goats and increased calcitriol concentrations in Ca reduced fed goats (Table 4) it can be speculated that in ruminants, in contrast to monogastric species, CDH17 expression was not diminished by calcitriol. Currently, there is no suitable assay available for measuring PTH concentrations in caprine plasma. Therefore, cAMP was measured as an indirect assessment for PTH response as it was done in human studies [34]. Increased cAMP plasma concentrations during dietary Ca reduction indicated a physiological response to temporarily diminished plasma Ca concentrations which probably occurred during this dietary intervention to stimulate renal calcitriol synthesis [35]. In contrast, cAMP concentrations in goats on dietary N reduction were not affected, indicating a missing increase in PTH secretion in response to diminished plasma Ca concentrations [35]. A potential modulator of CDH17 RNA expression in goats in the present study could be the Ca sensing receptor (CaR), which is also expressed in caprine intestinal epithelia [5]. Activation of the CaR by Ca stimulated the secretion of a signaling protein called wingless-type mouse mammary tumour virus integration site family, member 5A (WntA5) and its receptor (receptor tyrosine kinase-like orphan receptor 1, Ror1) in intestinal epithelial cell culture [36]. The WntA5 in turn stimulated expression of the transcription factor caudal type homeobox 2 (CDX2) [36] which was able to increase CDH17 mRNA expression in kidney cell culture [22]. It is conceivable that reduced plasma Ca concentrations in N reduced fed goats in the current study led to a reduced activation of the CaR and hence to reduced CDH17 mRNA expression via WnTA5, Ror1 and CDX2.

Expression of OCLN was increased on an mRNA level in the mid jejunum in the (N-/Ca+) and decreased on an mRNA level and on a protein level in the mid jejunum of Ca reduced fed goats in the present study. In studies with canine kidney cells and mouse intestinal epithelia, reduction in OCLN expression by selective knock down caused an increase in permeability to monovalent cations [37] and macromolecules [15]. This might have also occurred in Ca reduced fed goats leading to higher paracellular permeability for Ca which is also supported by numerically highest unidirectional Ca flux rates from mucosal to serosal in this feeding group [5]. Vice versa, increased OCLN expression in the (N-/Ca+) might have impaired epithelial permeability, again supported by numerically lowest mucosal to serosal Ca flux rates in this group [5], contributing to reduced plasma Ca concentrations in this feeding group. Furthermore, reduced OCLN expression was associated with increased expression of CLDN2 in intestinal epithelia in vitro and in vivo [15] and was also seen in the mid jejunum of goats receiving the Ca reduced diet in the present study, indicating that OCLN plays a role in determining the composition of TJ in a regulatory manner.

The ZO1 protein couples between TJ proteins and the cytoskeleton due to its ability to bind members of the TJ including OLCN and CLDN [38–41] as well as components of the cytoskeleton, for example F-actin [38, 39] and hence controls paracellular permeability [42]. Therefore, increased expression of ZO1 on an mRNA and protein level in the mid jejunum of N reduced fed goats might have reflected increased expression of TJ proteins other than CLDN2, CLDN12 and OCLN which were not increased in these animals, in order to couple these proteins with the cytoskeleton and ensure intact TJ assembly. Furthermore, increased expression of ZO1 was associated with increased barrier function of corneal epithelial cells [43] as well as reduced permeability of porcine ileal epithelia [44]. Hence, increased expression of ZO1 due to dietary N reduction might have been a compensation, for example for decreased CDH17 expression to ensure intact barrier function of the intestinal epithelium during this feeding regime.

Considering both, electrophysiological properties and expression of TJ and AJ proteins in connection with paracellular Ca transport, changes in these parameters during the applied feeding regimes were mainly seen in the proximal and mid jejunum but not in the ileum in the present study. This is in accordance with own previous observations indicating that changes in transcellular Ca absorption due to altered expression of involved transporting structures during dietary N reduction are also mainly seen in the proximal and mid jejunum [5]. While dietary Ca reduction stimulated transcellular Ca absorption in the ileum of young goats [5], the ileum was not involved in changed expression of TJ and AJ and paracellular Ca transport, indicating that compensation of low dietary Ca intake was already sufficient by increasing trans- and paracellular Ca absorption in the more proximal parts of the small intestines.

In conclusion, the current study showed that in addition to transcellular Ca transport also paracellular Ca transport was altered during dietary N as well as Ca reduction in young goats in a segment-specific manner. Low dietary N intake was potentially associated with increased epithelial permeability and greater loss of Ca into the intestinal lumen, while dietary Ca reduction resulted in segment specifically increased expression of TJ proteins probably connected with enhanced paracellular Ca absorption in addition to the transcellular pathway.

Increased expression of CLDN2 and CLDN12 during dietary Ca reduction was probably mediated by high calcitriol levels in these goats, while modulation of TJ protein expression during dietary N reduction seems to be more complex and includes hormones and/or substrates other than calcitriol. In further studies the role of calcitriol as a potential mediator of TJ protein expression in young goats should be investigated in more detail and other TJ components such as tricellulin which is located at tricellular contact points and affects paracellular barrier [45] should be included.

## Supporting Information

S1 Table Dry matter, concentrate, N, Ca and P intake and feed efficiency of growing goats receiving an N and/or Ca reduced diet. DM, dry matter; n = number of animals; data have already been published by Elfers et al. [5].
(DOCX)
