# Supplementary material for: Expression of Tight Junction Proteins and Cadherin 17 in the Small Intestine of Young Goats Offered a Reduced N and/or Ca Diet
Source: PLoS One. 2016 Apr 27;11(4):e0154311. doi: 10.1371/journal.pone.0154311 (PMC4847856; doi:10.1371/journal.pone.0154311)
Supplement: S1 Table — DM, dry matter; n = number of animals; data have already been published by Elfers et al. [5]. (DOCX) [file pone.0154311.s001.docx]

**S1Table. Dry matter, concentrate, N, Ca and P intake and** **feed efficiency of growing goats receiving an N and/or Ca reduced diet.**

| **Item** | **N+/Ca+** | **N-/Ca+** | **N+/Ca-** | **N-/Ca-** |
| --- | --- | --- | --- | --- |
| n | 7 | 6 | 6 | 7 |
| DM intake (g/d) | 763 | 652 | 741 | 702 |
| Concentrate intake (g/d) | 691 | 614 | 661 | 642 |
| Feed efficiency (kg/kg) | 0.19 | 0.16 | 0.17 | 0.12 |
| N intake (g/d) | 21.19 | 7.71 | 20.85 | 7.69 |
| Ca intake (g/d) | 8.08 | 7.08 | 2.91 | 2.37 |
| P intake (g/d) | 3.11 | 2.66 | 2.97 | 2.73 |
